# Associations of Screen Time and Physical Activity With Body Mass Index in Early Adolescence: A Prospective Cohort Study

**DOI:** 10.1002/oby.70181

**Published:** 2026-03-31

**Authors:** Jason M. Nagata, Isaac Frimpong, Nathan D. Nguyen, Alexander Heuer, Christiane K. Helmer, Abubakr A. Al‐Shoaibi, Kyle T. Ganson, Alexander Testa, Erin E. Dooley, Kelley Pettee Gabriel, Fiona C. Baker, Holly C. Gooding

**Affiliations:** ^1^ Division of Adolescent and Young Adult Medicine, Department of Pediatrics University of California San Francisco California USA; ^2^ Factor‐Inwentash Faculty of Social Work, University of Toronto Toronto Ontario Canada; ^3^ Department of Management Policy and Community Health, University of Texas Health Science Center at Houston Houston Texas USA; ^4^ Department of Epidemiology University of Alabama at Birmingham Birmingham Alabama USA; ^5^ Center for Health Sciences, SRI International Menlo Park California USA; ^6^ Division of General Pediatrics and Adolescent Medicine, Department of Pediatrics Emory University School of Medicine Atlanta Georgia USA

**Keywords:** body composition, exercise, teen, wearable device, youth

## Abstract

**Objective:**

This study aimed to prospectively identify the independent, mutually adjusted, and interactive associations of screen time and physical activity with BMI and overweight/obesity risk in adolescents.

**Methods:**

This study analyzed prospective data from 5356 US adolescents in the Adolescent Brain Cognitive Development (ABCD) Study, Year 2 (2018–2020, ages 11–12) and Year 4 (2020–2022, ages 13–14).

**Results:**

Mean daily screen time was 6.1 (±5.2) hours and mean step count was 9265 (±3227) steps in Year 2; 32.7% were classified with overweight/obesity in Year 4. In Poisson regression models examining the exposures mutually adjusted for each other, high screen time (> 8 h/day) was associated with higher risk of overweight/obesity (risk ratio [RR], 1.09; 95% CI, 1.02–1.16; *p* = 0.013) compared with low screen time (0–4 h/day). In multivariable linear and Poisson regression models, respectively, low step count (1000–6000 steps/day) was associated with higher BMI percentile (coefficient [*B*], 3.27; 95% CI, 1.54–4.99; *p* < 0.001) and higher risk of overweight/obesity (RR, 1.23; 95% CI, 1.07–1.40; *p* = 0.003) compared with high step count (> 12,000 steps/day). No significant interactions were observed between screen time and step count.

**Conclusions:**

These findings suggest that obesity prevention efforts should consider both greater physical activity and less recreational screen use as behavioral interventions.

## Study Importance

- What is already known?
  ○ Prolonged sedentary behavior and low physical activity have been linked to higher BMI and increased risk of overweight and obesity in adolescents. The *2018 Physical Activity Guidelines Advisory Committee Scientific Report* highlighted the need for further research on the impact of dose–response relationships of screen time and physical activity on adolescent health outcomes.
- What does this study add?
  ○ In this prospective study of 5356 US adolescents, high screen time (> 8 h per day) and low objectively measured physical activity (1000–6000 steps per day) were independently associated with overweight and obesity and remained significant when mutually adjusted for in the same model.
- How might these results change the direction of research or the focus of clinical practice?
  ○ These findings can inform physical activity and screen time guidelines for early adolescents and suggest that obesity prevention efforts should consider both targets of increasing physical activity and decreasing recreational screen use as behavioral interventions.

## 1 Introduction

Daily physical activity is associated with improvements in adolescent body composition [1], self‐esteem [2], and mental health [3]. The 2018 *Physical Activity Guidelines for Americans*, established by the United States (US) Department of Health and Human Services, advise that children and adolescents (ages 6–17) engage in at least 60 min of moderate‐to‐vigorous intensity physical activity (MVPA) daily [4]. Previous studies show that less than 25% of US children and adolescents meet this recommendation [5, 6]. Simultaneously, as recreational screen time among adolescents continues to rise, it may increasingly encroach upon time that could be spent engaging in physical activity [7]. In 2016, US adolescents averaged 4–6 h per day spent on digital media [8, 9]. In 2024, the US Surgeon General's Advisory on Social Media and Youth Mental Health reported that a quarter of adolescents engage in greater than 5 h of screen time per day [10].

Low physical activity and prolonged sedentary behavior, including excessive screen time, have both been linked to negative effects on adolescent weight status, including higher body mass index (BMI) and increased risk of overweight and obesity [11, 12]. While BMI is an imperfect measure for adiposity, it remains the most widely used and accessible indicator for identifying excess weight and estimating obesity risk. Studies have shown that elevated BMI in adolescence is associated with long‐term health consequences such as metabolic syndrome, diabetes, and cardiovascular disease in adulthood [13, 14]. A meta‐analysis in 2016 revealed that individuals with obesity during adolescence were five times more likely to have obesity in adulthood [15]. Given that lifestyle behaviors, overweight, and obesity during adolescence often persist into later life, this period demonstrates a critical window for early intervention [16, 17].

The *2018 Physical Activity Guidelines Advisory Committee Scientific Report* highlights the need for further research on the dose–response relationship and interactive effects between screen time and physical activity in adolescents, as well as for more longitudinal studies in this area [18]. It also noted that current research on sedentary behavior and health is constrained by a dearth of studies using device‐based measures of sedentary time and that previous studies are limited by reliance on proxy measures, particularly television viewing, which confound true sedentary time with other exposures (e.g., advertising, eating behaviors) [18]. Similarly, research recommendations from the 2020 WHO Physical Activity and Sedentary Behavior Guidelines Development Group underscored the need to better understand dose–response patterns and interactive effects between physical activity and sedentary behavior across age groups [17]. Despite these calls, there remains a lack of prospective research examining these relationships in children and adolescents, leaving the prospective dose–response and joint effects of physical activity and sedentary behavior, including screen time, poorly understood [17]. This evidence gap limits the development of appropriate, dose–specific directives for physical activity and screen time in adolescents. Further investigation is needed to inform the development of early interventions and guidelines to improve adolescent health and help prevent overweight and obesity.

Most existing studies examining screen time and physical activity in relation to BMI have used cross‐sectional designs, with mixed findings regarding their interaction and joint associations [19, 20]. Few recent studies have explored how sedentary behavior and physical activity jointly predict BMI over time in adolescence; these studies generally suggest that increases in sedentary behavior and declines in physical activity are prospectively associated with higher BMI [21, 22, 23, 24, 25]. However, most of these studies did not specifically consider screen time in their sedentary behavior measure. A recent prospective study in German children that specifically included screen time observed that reductions in screen time, combined with increases in physical activity, were associated with favorable within‐person BMI changes 1 year later [25]. An earlier longitudinal review also examined physical activity and sedentary behavior (e.g., reading, talking with friends, and using the internet) in relation to weight gain in youth [26]. However, this review was published in 2005, before the dramatic increase and transformation of screen time among adolescents, and did not capture contemporary modalities such as social media, texting, video chat, and streaming platforms that now characterize adolescent screen use. Therefore, our study provides a more current perspective by including modern forms of screen engagement.

Using data from the Adolescent Brain Cognitive Development (ABCD) Study, our previous cross‐sectional analysis found that combinations of medium/high screen time and low step counts were associated with higher BMI percentiles in early adolescence [20]. The current analysis builds on these findings by leveraging the ABCD Study's prospective study design with 2‐year follow‐up that characterizes a critical window for development of overweight and obesity, allowing for better delineation of temporal relationships and further analysis of the joint associations of physical activity (daily step count) and sedentary behavior (screen time) exposures with BMI percentile and overweight/obesity risk. The study also addresses gaps in evidence on potential dose–specific associations by examining how different levels of exposure to screen time (i.e., hours per day) and physical activity (i.e., steps per day) relate to BMI percentile and overweight/obesity risk.

## 2 Methods

### 2.1 Study Procedures and Participants

We used data from the ABCD Study Year 2 (2018–2020) and Year 4 (2020–2022) follow‐up assessments to address the study objectives. The ABCD Study was initiated between 2016 and 2018 (baseline) and is the largest sustained assessment of brain development and adolescent health in the US. The study recruited 11,875 children from 21 sites across the US. Previous investigations have detailed the study's design, recruitment methods, data collection, and procedures [27]. Out of the 9450 participants who completed both Year 2 and Year 4 assessments, 2872 were missing Fitbit data, 67 were missing screen time data from Year 2, and 1155 were missing Year 4 BMI data, resulting in a final sample size of 5356 participants (Table S1). Institutional review board (IRB) approval was obtained at the University of California, San Diego (UCSD) and at each study site. Participants provided written assent, and caregivers provided written informed consent.

### 2.2 Exposure Variables (Year 2)

All exposure data were extracted from the ABCD Study Year 2 follow‐up (November 2018–November 2020).

#### 2.2.1 Screen Time

Self‐reported screen time data were collected via the ABCD Youth Screen Time Survey, which asked adolescents to estimate the average number of hours they spend using screens recreationally (e.g., watching television and videos, playing video games, texting, social media, video chat) on weekdays and weekends, separately. After summing the time spent across each modality, the total screen use weighted average was calculated with the formula: ([weekday average × 5] + [weekend average × 2])/7. Screen time was categorized as 0–4 h (low), 4–8 h (medium), or greater than 8 h (high). This categorization was based on prior studies identifying 4 h per day as a threshold linked to cardiovascular risk factors and metabolic dysfunction in adolescents [20, 28] and aligns with other national surveys using similar cutoffs [20, 29].

#### 2.2.2 Daily Steps (Fitbit)

Participants enrolled in the ABCD Study were invited to participate in Fitbit‐based data collection (Fitbit Charge series device, Fitbit Inc.) for a period of 21 days, in which their daily steps were measured. Of the 7050 participants enrolled in the optional Fitbit‐based physical activity assessment during Year 2, 5356 had complete data on Fitbit, sociodemographic, and other relevant study variables and were included in the final analytic sample. Studies have shown the validity and feasibility of utilizing Fitbit devices to evaluate physical activity and daily step counts over time in adolescent populations [30]. Data were retrieved and interpreted using the ABCD Study guidelines [27, 30, 31]. Based on recommendations from previous studies, only days with greater than 599 min of waking wear time and greater than 1000 steps were included in the analysis. The data were further processed by grouping daily steps into low (1000–6000 steps), medium (6000–12,000 steps), and high (greater than 12,000 steps). These categorizations were based on the physical activity standards for adolescents which recommend 60 min of MVPA per day [4]. A total of 12,000 steps meets these recommendations [32], and 6000 steps represents a half‐dose of physical activity [33].

### 2.3 Outcome Variables (Year 4)

All outcome data were extracted from the ABCD Study Year 4 follow‐up (November 2020–November 2022).

Participants' weight and height were individually measured at study locations utilizing the Health‐o‐meter 844KL High‐Capacity Digital Bathroom Scale and a carpenter's square steel tape measure. The formula weight (kg)/height (m)^2^ was used to calculate BMI, which was then converted into percentiles based on age and sex. Participants with BMI between the 85th and 95th percentiles were classified as overweight, whereas those with BMI at or above the 95th percentile were classified as having obesity [34], in accordance with guidelines from the Centers for Disease Control and Prevention [35]. A binary composite variable was subsequently created for overweight or obesity (BMI ≥ 85th percentile).

### 2.4 Covariates

Previous studies have shown that sex, racial and ethnic background, socioeconomic status, parent education level, and parent marital status are associated with screen time, physical activity, and BMI [9, 36, 37]. Covariates included in the present analysis were Year 2 participant age, sex (female or male), race and ethnicity (Asian, Black, Latino/Hispanic, Native American, White, or other), household income (two categories reflecting the US median household income: less than $75,000 and $75,000 or more [38]), parental education status (high school education or less vs. college education or higher), and parental marital status (married/partnered vs. single/separated). COVID‐19 pandemic onset was also included as a covariate as data collection may have been influenced by the COVID‐19 pandemic during Year 2 data collection. March 13, 2020, was considered as the pandemic onset and dates of Fitbit data were analyzed accordingly (before vs. during pandemic). Participants whose monitoring periods began before and extended into the pandemic were also classified as “during pandemic.” Finally, BMI percentile at Year 2 was also included as a covariate in all prospective models.

### 2.5 Statistical Analysis

Stata 18 (StataCorp LLC) was utilized for data analysis. First, we calculated descriptive statistics from the analytical study sample. Multivariable linear regression was used to determine screen time and step count associations with BMI. Modified Poisson regression was used to determine screen time and step count associations with overweight/obesity [39]. Three models were run for each exposure variable (continuous and categorical) measured at Year 2 to predict BMI outcomes at Year 4. Model 1 assessed screen time alone at Year 2, Model 2 examined steps alone at Year 2, and Model 3 included both screen time and steps at Year 2, mutually adjusted for each other. All statistical models were adjusted for age, sex, race and ethnicity, household income, parent education levels, parent marital status, COVID‐19 data collection period, calendar month of data collection, and BMI percentile at Year 2. To assess a linear dose–response relationship, we used linear and Poisson regression with the exposures modeled as numeric predictors, such that estimates reflect a one‐category increase in screen time or step count. We also tested for interaction between step count and screen time on BMI percentile and overweight/obesity by including a product term (steps/day × screen time/day). To assess the joint effects of screen time and physical activity, we created a comprehensive nine‐category exposure variable by cross‐classifying all screen time categories (0–4, 4–8, > 8 h daily) with all step count categories (1000–6000, 6000–12,000, > 12,000 steps/day). This approach allowed us to examine associations across the full spectrum of recreational screen time and physically active behaviors. Significance was indicated by a two‐sided *p* value < 0.05.

## 3 Results

Among 5356 adolescents included in this analysis (mean age at Year 2: 12.0 (±0.6) years), 49.6% were female and 39.0% were from racial/ethnic minority groups. Adolescents reported a mean recreational screen time of 6.1 (±5.2) hours per day and had an overall mean step count of 9265 (±3227) steps per day over the course of 21 days at Year 2. Nearly a third (32.7%) of adolescents were classified with overweight or obesity at Year 4 (Table 1). Additional sample characteristics at Year 4 can be found in Table S2.

**TABLE 1:** Sample characteristics of Adolescent Brain Cognitive Development (ABCD) Study participants at Year 2 included in the current analyses (*N* = 5356).

Sociodemographic characteristics	Mean (SD)/*n* (%)
Age (years)	12.0 (0.6)
Sex	
Female	2656 (49.6%)
Male	2700 (50.4%)
Race and ethnicity	
Asian	284 (5.3%)
Black	643 (12.0%)
Latino/Hispanic	926 (17.3%)
Native American	166 (3.1%)
White	3267 (61.0%)
Other	70 (1.3%)
Household income	
Less than $75,000	2367 (44.2%)
$75,000 or more	2989 (55.8%)
Parent education	
High school education or less	611 (11.4%)
Some college education or more	4745 (88.6%)
Parent marital status	
Parent married/partnered	3952 (73.8%)
Parent not married/unpartnered	1404 (26.2%)
Physical activity variables (Fitbit‐derived)	
Total steps per day	9265 (3227)
Step categories (steps/day)	
1000–6000 (low)	985 (18.4%)
6000–12,000 (medium)	3616 (67.5%)
> 12,000 steps per day (high)	755 (14.1%)
Self‐reported screen time	
Total recreational screen time (h/day)	6.1 (5.2)
Screen time categories (h/day)	
0–4 (low)	2406 (44.9%)
4–8 (medium)	1623 (30.3%)
> 8 (high)	1327 (24.8%)
Anthropometric measures (Year 4)	
BMI percentile	64.6 (29.6)
Overweight or obesity (≥ 85th percentile)	1749 (32.7%)

In regression models examining screen time at Year 2, not adjusted for step count (Model 1 in Table 2), high screen time (> 8 h/day) was prospectively associated with higher risk of overweight or obesity at Year 4 (risk ratio [RR], 1.08; 95% confidence interval [CI], 1.01–1.16) compared with the low screen time (0–4 h/day) category. A dose–response relationship was observed, with overweight or obesity risk increasing across screen time categories (RR, 1.04; 95% CI, 1.00–1.07).

**TABLE 2:** Associations between self‐reported total recreational screen time and Fitbit step count categories and BMI outcomes in the Adolescent Brain Cognitive Development (ABCD) Study (*N* = 5356).

	Model 1^a^, ^b^		Model 2^a^, ^c^		Model 3^a^, ^d^	
	BMI percentile		BMI percentile		BMI percentile	
	*B* (95% CI)	*p*	*B* (95% CI)	*p*	*B* (95% CI)	*p*
Screen time (h/day)						
0–4 (low)	Reference		NA	NA	Reference	
4–8 (medium)	−0.17 (−1.26 to 0.92)	0.759	NA	NA	0.03 (−1.06 to 1.12)	0.951
> 8 (high)	0.63 (−0.59 to 1.85)	0.311	NA	NA	0.97 (−0.26 to 2.21)	0.123
Physical activity (steps/day)						
1000–6000 (low)	NA	NA	**3.09 (1.38 to 4.80)**	**< 0.001**	**3.27 (1.54 to 4.99)**	**< 0.001**
6000–12,000 (medium)	NA	NA	1.23 (−0.15 to 2.62)	0.081	1.35 (−0.04 to 2.74)	0.057
> 12,000 (high)	NA	NA	Reference		Reference	
Continuous						
Screen time, h/day	0.01 (−0.08 to 0.10)	0.840	NA	NA	0.03 (−0.06 to 0.13)	0.504
Physical activity (1000 steps/day)	NA	NA	**0.23 (0.08 to 0.39)**	**0.003**	**0.24 (0.09 to 0.39)**	**0.002**

	Overweight or obesity		Overweight or obesity		Overweight or obesity	
	Risk ratio (95% CI)	*p*	Risk ratio (95% CI)	*p*	Risk ratio (95% CI)	*p*
Screen time (h/day)						
0–4 (low)	Reference		NA	NA	Reference	
4–8 (medium)	1.02 (0.96 to 1.10)	0.480	NA	NA	1.03 (0.96 to 1.10)	0.389
> 8 (high)	**1.08 (1.01 to 1.16)**	**0.020**	NA	NA	**1.09 (1.02 to 1.16)**	**0.013**
Physical activity (steps/day)						
1000–6000 (low)	NA	NA	**1.33 (1.16 to 1.52)**	**< 0.001**	**1.23 (1.07 to 1.40)**	**0.003**
6000–12,000 (medium)	NA	NA	**1.14 (1.04 to 1.26)**	**0.007**	1.09 (0.99 to 1.21)	0.073
> 12,000 (high)	NA	NA	Reference		Reference	
Continuous						
Screen time, h/day	1.00 (1.00 to 1.01)	0.121	NA	NA	1.00 (1.00 to 1.01)	0.128
Physical activity (1000 steps/day)	NA	NA	**0.96 (0.95 to 0.98)**	**< 0.001**	**0.96 (0.95 to 0.98)**	**< 0.001**

*Note*: Bold values indicate statistical significance.

Abbreviation: NA, not applicable.

^a^ All models adjusted for Year 2 BMI percentile, sex, race/ethnicity, household income, parent education, parent marital status, and data collection period (pre‐COVID‐19, during COVID‐19).

^b^ Model 1: Screen time categories only.

^c^ Model 2: Step count categories only.

^d^ Model 3: Screen time and step count categories mutually adjusted for each other.

In regression models examining step count categories at Year 2, not adjusted for screen time (Model 2 in Table 2), the low step count category (1000–6000 steps/day) was prospectively associated with higher BMI percentile at Year 4 (coefficient [*B*], 3.09; 95% CI, 1.38–4.80) compared with the high step count category. Tests for linear trend confirmed a dose–response relationship, with each one‐category increase in step count prospectively associated with a 1.67‐point lower BMI percentile at Year 4 (95% CI, −2.52 to −0.81). The low (1000–6000 steps/day) and medium (6000–12,000 steps/day) step count categories were prospectively associated with higher risk of overweight or obesity at Year 4 (low: RR, 1.33; 95% CI, 1.16–1.52; medium: RR, 1.14; 95% CI, 1.04–1.26) compared with the high step count category.

In regression models including both screen time and step count categories at Year 2, mutually adjusted for each other (Model 3 in Table 2), high screen time (> 8 h/day) remained significantly associated with overweight or obesity risk at Year 4 (RR, 1.09; 95% CI, 1.02 to 1.16). The low step count category (1000–6000 steps/day) at Year 2 was prospectively associated with higher BMI percentile at Year 4 (*B*, 3.27; 95% CI, 1.54 to 4.99) and a higher overweight or obesity risk at Year 4 (RR, 1.23; 95% CI, 1.07 to 1.40) compared with the high step count category. In the mutually adjusted model, a dose–response relationship was observed across step count categories, with BMI decreasing as step count category increased (*B*, −1.72; 95% CI, −2.59 to −0.86). A dose–response relationship between screen time and overweight/obesity was also observed, with each one‐category increase in screen time prospectively associated with a 4% higher risk of overweight/obesity (RR, 1.04; 95% CI, 1.01 to 1.08). When screen time, step count, and BMI percentile were all assessed as continuous variables, screen time was not significantly associated with BMI percentile and physical activity was associated with greater BMI percentile (*B*, 0.24; 95% CI, 0.09 to 0.39) (Table 2). Similar associations were observed for overweight/obesity risk when examining continuous physical activity: RR, 0.96; 95% CI, 0.95 to 0.98 (Table 2). Physical activity showed stronger and more consistent associations with both BMI and overweight/obesity outcomes at Year 4 compared to screen time (Table 2).

No significant interactions were observed between screen time and step count categories for BMI percentile (*p* for interaction = 0.45) or overweight/obesity outcomes (*p* for interaction = 0.38), indicating that their effects were independent and additive rather than synergistic.

**FIGURE 1:**
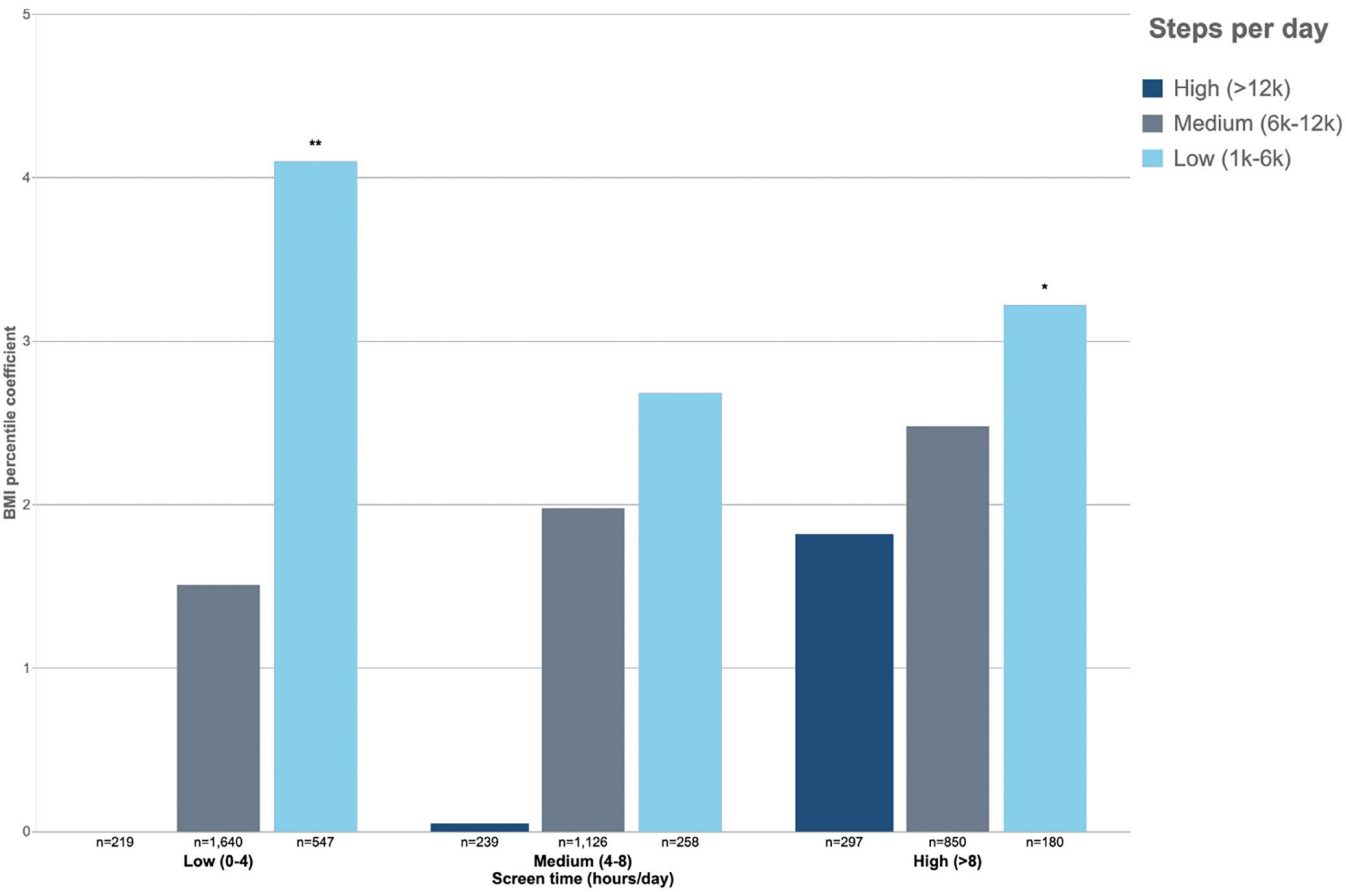
Prospective joint associations between screen time and step count category at Year 2 and BMI percentile at Year 4 in 5356 participants of the ABCD Study. The reference category is low screen time/high steps (*n* = 219). Asterisks indicate statistical significance: *p < 0.05, **p < 0.01. [Color figure can be viewed at wileyonlinelibrary.com]

In the joint screen time and step count analysis, significant associations were observed for low screen time/low step count (*B*, 4.10; *p* < 0.01) and high screen time/low step count (*B*, 3.27; *p* < 0.05) (Figure 1). While not all findings were significant, categories involving low step count consistently showed higher BMI percentiles at Year 4, regardless of screen time level (Figure 1).

## 4 Discussion

In this prospective study of a large national sample of US adolescents from the ABCD Study, we found that higher screen time and lower step count were associated with greater risk of overweight or obesity 2 years later. We specifically found a dose–response relationship between higher screen time category and increased overweight/obesity risk as well as higher step count category and lower BMI percentile. These results build upon our prior cross‐sectional analysis of ABCD Study data from Year 2, which showed higher BMI percentile and overweight or obesity risk with higher screen time and lower step count [20]. Unlike the previous study, the present prospective analysis did not find a significant interaction between screen time and step count for BMI percentile, and the association for screen time was not observed for continuous BMI percentile. By jointly examining both behaviors over time, our study underscores the importance of addressing not only sedentary screen time but also promoting physical activity as intervention targets to support healthy development in adolescence.

These findings are consistent with prior prospective research examining physical activity, sedentary behavior, and BMI. Longitudinal studies in children and adolescents have shown that declines in physical activity and increases in sedentary behavior, including screen time, are associated with higher BMI over time [21, 22, 23, 24]. Similarly, a recent study observed that reductions in screen time in addition to increased physical activity were associated with favorable BMI changes, supporting our observation that both behaviors independently contribute to overweight and obesity risk [25]. While several prior studies have used objectively measured step counts, our study extends this work by using objective step count data from the large US national sample of the ABCD Study, allowing for a robust prospective examination of the effects of both screen time and physical activity on BMI and overweight or obesity risk in adolescents.

The association between high screen time (> 8 h/day) and risk of overweight or obesity remained statistically significant even after adjusting for objectively measured physical activity. This suggests that screen time may contribute to excess adiposity through behavior or metabolic mechanisms not fully mitigated by physical activity, such as disrupted sleep, exposure to food marketing, or increased snacking [20, 40]. In our models, screen time was not significantly associated with BMI percentile when treated as a continuous outcome, but it was associated with the categorical classification of overweight or obesity. This finding suggests that the relationship may not be linear across the entire BMI distribution. Instead, higher screen time may increase the likelihood of crossing the threshold into the overweight or obesity category without proportionately raising BMI percentile across all ranges. This finding is consistent with prior research that found associations between screen time and adiposity indicators such as higher skinfold thickness, fat mass index, and insulin resistance after adjusting for BMI and physical activity [40].

Low step count (1000–6000 steps/day) was associated with both higher BMI percentile and greater risk of overweight or obesity at follow‐up, even after adjusting for screen time. While medium step count (6000–12,000 steps/day) was also associated with a higher risk in physical activity‐only models, this association was attenuated in models mutually adjusted for screen time and step count. These findings support prior literature suggesting a possible dose–response relationship between step count and the risk of overweight or obesity [20, 41, 42, 43, 44]. Our findings also suggest that maintaining a higher step count may offer additional protection against excess weight gain. Notably, the associations for step count with both BMI percentile and overweight or obesity were generally stronger and more consistent compared to screen time, underscoring the critical role of physical activity in healthy weight development during adolescence.

We did not find significant interaction between physical activity and screen time, indicating that high activity levels may not fully offset the negative effects of prolonged screen use, and vice versa, on overweight/obesity risk. However, the joint, combined category models revealed important behavioral patterns. Adolescents with both high screen time (> 8 h/day) and low step counts (1000–6000 steps/day) were most likely to exhibit higher BMI. These findings align with previous cross‐sectional research indicating that the protective effects of physical activity may have an upper limit in the context of excessive sedentary screen use [20]. Given that adolescence is a critical developmental period for establishing long‐term health behaviors [13, 14], these results underscore the importance of promoting both increased physical activity and reduced screen time to mitigate obesity risk during adolescence.

This study has several strengths. First, it includes a large population‐based sample, enhancing statistical power. Second, the use of objectively measured physical activity data collected over 21 consecutive days provides a more reliable estimate of daily movement than self‐reported activity. Third, the prospective nature of our study design with a 2‐year follow‐up period allowed us to establish temporal associations between screen time and step count and subsequent BMI percentile and status. While some physical activity and screen time may have changed during the follow‐up period, particularly due to the onset of the COVID‐19 pandemic, this prospective design helps ensure the temporal ordering of variables and reduces the risk of reverse causation.

However, this study also has limitations. Activity data were limited to a 3‐week window and may not fully capture annual physical activity patterns or seasonal variation. Screen time was self‐reported and subject to recall bias and misreporting. Additionally, the measure did not capture content exposure, which may differentially affect obesity risk [9]. In addition, BMI percentile was used as the primary outcome, but it cannot distinguish between fat and lean mass. Future studies may consider using a more precise measure of adiposity or metabolic risk. Another limitation is that sociodemographic differences were observed between participants included in the analysis and those excluded due to missing data. Finally, there is a potential for unexamined confounders, although we controlled for sociodemographic factors and the onset of the COVID‐19 pandemic.

## 5 Conclusion

This prospective study contributes to the growing body of literature highlighting the independent and joint effects of step count and screen time on adolescent BMI. Among a large national cohort, adolescents with a step count below 12,000 steps per day and screen time exceeding 8 h per day were at the greatest risk for overweight and obesity. The findings suggest that both higher physical activity and lower recreational screen use are important targets for obesity prevention efforts in early adolescence. Prevention strategies and behavioral interventions should consider both behavioral targets. Future research should include a more precise measure of adiposity or metabolic risk and long‐term objective data and should examine physical activity and screen time trajectories to further inform physical activity and screen time guidelines for early adolescents.

## Author Contributions

**Jason M. Nagata:** supervision, formal analysis, data curation, conceptualization, writing – original draft, writing – review and editing. **Isaac Frimpong:** formal analysis, writing – original draft, writing – review and editing. **Nathan D. Nguyen:** formal analysis, writing – original draft, writing – review and editing. **Alexander Heuer:** formal analysis, writing – original draft, writing – review and editing. **Christiane K. Helmer:** formal analysis, writing – original draft, writing – review and editing. **Abubakr A. Al‐Shoaibi:** formal analysis, writing – original draft, writing – review and editing. **Kyle T. Ganson:** writing – review and editing. **Alexander Testa:** writing – review and editing. **Erin E. Dooley:** writing – review and editing. **Kelley Pettee Gabriel:** writing – review and editing. **Fiona C. Baker:** writing – review and editing, conceptualization. **Holly C. Gooding:** writing – review and editing. All authors approved the final manuscript as submitted and agree to be accountable for all aspects of the work.

## Funding

This work was supported by the National Institutes of Health (K08HL159350) and the Doris Duke Charitable Foundation (2022056). The funders had no role in the design and conduct of the study; collection, management, analysis, and interpretation of the data; preparation, review, or approval of the manuscript; and decision to submit the manuscript for publication. The ABCD Study was supported by the National Institutes of Health and additional federal partners under award numbers U01DA041022, U01DA041025, U01DA041028, U01DA041048, U01DA041089, U01DA041093, U01DA041106, U01DA041117, U01DA041120, U01DA041134, U01DA041148, U01DA041156, U01DA041174, U24DA041123, and U24DA041147. A full list of supporters is available at https://abcdstudy.org/federal‐partners/. A listing of participating sites and a complete listing of the study investigators can be found at https://abcdstudy.org/principal‐investigators.html. ABCD consortium investigators designed and implemented the study and/or provided data but did not necessarily participate in the analysis or writing of this report.

## Conflicts of Interest

The authors declare no conflicts of interest.

## Supporting information


**Table S1:** Comparison of the sociodemographic characteristics of the Adolescent Brain Cognitive Development (ABCD) Study participants included versus excluded in the analysis.
**Table S2:** Sample characteristics of Adolescent Brain Cognitive Development (ABCD) Study participants at Year 4 included in the current analyses (*N* = 5356).

## Data Availability

Data used in the preparation of this article were obtained from the ABCD Study (https://abcdstudy.org), held in the NIH Brain Development Cohorts (NBDC) Portal.
