# Supplementary material for: Associations of Screen Time and Physical Activity With Body Mass Index in Early Adolescence: A Prospective Cohort Study
Source: Obesity (Silver Spring). 2026 Mar 31;34(5):1092–100. doi: 10.1002/oby.70181 (PMC13116022; doi:10.1002/oby.70181)
Supplement: Supplementary file 1 — Table S1: Comparison of the sociodemographic characteristics of the Adolescent Brain Cognitive Development (ABCD) Study participants included versus excluded in the analysis. Table S2: Sample characteristics of Adolescent Brain Cognitive Development (ABCD) Study participants at Year 4 included in the current analyses (N = 5356). [file OBY-34-1092-s001.docx]

**Table S1. Comparison of the sociodemographic characteristics of the Adolescent Brain Cognitive Development (ABCD) Study participants included vs. excluded in the analysis**

| Sociodemographic characteristics | Included  (n=5,356) | Excluded  (n=6,606) | p |
| --- | --- | --- | --- |
| Age (years) | 12.1 (0.7) | 11.9 (0.7) | 0.102 |
| Sex |  |  | 0.310 |
| Female | 48.4% | 47.4% |  |
| Male | 51.6% | 52.6% |  |
| Race and ethnicity |  |  | **<0.001** |
| Asian | 5.4% | 6.5% |  |
| Black | 15.1% | 25.0% |  |
| Latino/Hispanic | 14.6% | 19.6% |  |
| Native American | 3.7% | 3.3% |  |
| Other | 0.7% | 1.1% |  |
| White | 60.5% | 44.5% |  |
| Household income |  |  | **<0.001** |
| Less than $75,000 | 22.2% | 36.9% |  |
| $75,000 or more | 77.8% | 63.1% |  |
| Parent's highest education |  |  | **<0.001** |
| High school education or less | 9.2% | 18.7% |  |
| College education or more | 90.8% | 81.3% |  |
| Parent's marital status |  |  | **<0.001** |
| Parent married/partnered | 78.4% | 70.1% |  |
| Parent not married/unpartnered | 21.6% | 29.9% |  |

| **Table S2. Sample characteristics of Adolescent Brain Cognitive Development (ABCD) Study participants at Year 4 included in the current analyses (N = 5,356)** | |
| --- | --- |
| Sociodemographic characteristics | Mean (SD) / n (%) |
| Age (years) | 14.2 (0.7) |
| Household income |  |
| Less than $75,000 | 1,383 (27.1%) |
| $75,000 or more | 3,725 (72.9%) |
| Parent education |  |
| High school education or less | 465 (8.7%) |
| Some college education or more | 4,889 (91.3%) |
| Parent marital status |  |
| Parent married/partnered | 4,182 (78.3%) |
| Parent not married/unpartnered | 1,157 (21.7%) |
| Self-reported screen time (hours) |  |
| Total recreational screen time | 9.3 (6.1) |
| Screen time categories (h/d) |  |
| 0 to 4 (low) | 1,085 (20.3%) |
| 4 to 8 (medium) | 1,686 (31.5%) |
| >8 (high) | 2,575 (48.2%) |
| Anthropometric measures |  |
| BMI percentiles | 64.6 (29.6) |
| Overweight or obesity (≥85th percentile) | 1,749 (32.7%) |
| Due to missing data, not all category totals sum to 5,356. SD = standard deviation | |
